# Factors associated with successful psychiatric intensive home treatment – a quasi-experimental study

**DOI:** 10.3389/fpsyt.2026.1871702

**Published:** 2026-08-24

**Authors:** Stefan Weinmann, Konstantinos Nikolaidis, Gerhard Längle, Sebastian von Peter, Peter Brieger, Juergen E. Timm, Lasse Fischer, Morten Dreher, Svenja Raschmann, Martin Holzke, Julian Schwarz, Olaf Hardt, Constance Hirschmeier, Uwe Herwig, Stefan Klingberg, Reinhold Kilian, Johanna Baumgardt, Johannes Hamann, Andreas Bechdolf

**Affiliations:** 1 University of Lübeck, Lübeck, Germany; 2 Center of Integrative Psychiatry, Lübeck, Germany; 3 Department of Psychiatry, Psychotherapy, and Psychosomatics, incorporating FRITZ am Urban and Soulspace, Vivantes Hospital am Urban and Vivantes Hospital im Friedrichshain, Berlin, Germany; 4 Department of Psychiatry and Psychotherapy, Charité Campus Mitte, Charité – Universitätsmedizin Berlin, corporate member of Freie Universität Berlin and Humboldt-Universität zu Berlin, Berlin, Germany; 5 German Center for Mental Health, Berlin–Potsdam Site, Berlin, Germany; 6 Kbo-Isar-Amper-Klinikum Haar, Haar, Germany; 7 Universitat Bremen, Bremen, Germany; 8 Vivantes Klinikum Neukolln, Berlin, Germany; 9 Universitat Konstanz Zentrum fur Psychiatrie Reichenau, Reichenau, Germany; 10 Eberhard Karls Universitat Tubingen, Tübingen, Germany; 11 Universitat Ulm, Ulm, Germany; 12 Wissenschaftliches Institut der AOK, Bonn, Germany; 13 Bezirkslinikum Mainkofen, Deggendorf, Germany

**Keywords:** home treatment, mental health systems, predictors, psychosocial health, severe mental illness

## Abstract

**Background:**

With more and more Home Treatment (HT) teams being implemented worldwide as well as high variations in models and intensity of care and target groups, there is a need to look at factors associated with its success and relative effectiveness compared to inpatient treatment (IT). For doing so, we used data from the quasi-experimental evaluation of the German highly regulated model of intensive HT (IHT).

**Methods:**

Within a propensity-score matched study we compared 200 patients treated by IHT in ten psychiatric centers to 200 inpatients and assessed inpatient re-admission rate and days spent in hospital within the 12-months follow-up time. We used a set of clinical and sociodemographic variables within a multivariate regression model to evaluate the influence of variables affecting the relative effectiveness of IHT versus IT.

**Results:**

Controlling for diagnosis, age, treatment history, marital and employment status, IHT was more effective than IT for women compared to men in avoiding readmissions. Furthermore, IHT was more effective for people with less impairment and those with either no inpatient treatment history or a high number of previous hospitalizations in the last two years.

**Conclusion:**

IHT might be more beneficial in women due to their stronger preference or advantages in involving community resources among women. IHT might be suitable for those with less severe psychiatric crises but also for high utilizers to stop revolving door patterns.

## 1 Introduction

In many mental health systems and regions, alternatives for hospitalization during acute psychiatric crises have been implemented and evaluated (1, 2). In particular, the evidence for Home Treatment (HT) or Crisis Resolution Team (CRT) treatment (Department of Health and Social Care (3), has been growing. In particular, the evidence for Home Treatment (HT) or Crisis Resolution Team (CRT) treatment has been growing (4), crisis teams have gatekeeping functions whereas in others, HT is only one option within a range of services offered. Recently, in a variety of European countries such as Switzerland (5) and the Netherlands (6), controlled studies conducted in the last years have shown that patients treated by a multidisciplinary team outside the hospital, have spent less time in inpatient settings during one year. Results with regard to hospital admission rates, however, have been mixed. With clinical as well as psychosocial outcomes and quality of life being comparable between HT and inpatient treatment (7), most studies demonstrated that HT is a viable alternative for hospitalization.

HT, however, accounts for only a small number of treatment episodes in people with serious crises requiring otherwise hospital admission. Research in the last years has focused (a) on policies to facilitate the establishment of such teams (2), (b) on HT/CRT implementation strategies to unravel their full potential (8, 9), and (c) on the identification of target groups which may benefit most. Notably, whereas the early British (7) and Australian (10) studies primarily included people with a diagnosis of a psychotic disorder, more recently published European studies evaluated HT models which included more patients with affective disorders (5, 11, 12).

To respond to critical voices expressing concerns towards HT (13, 14), models must be evaluated continuously against modern inpatient control conditions in a variety of mental health systems. In the fragmented mental health care system in Germany (15), for example, HT has until recently only been available in regional pilot integrated care projects depending on selected health insurance companies’ willingness to pay annual budgets to groups of patients leaving it up to the mental health system to decide on the mode of care (16) Only recently, since 2018, it has by law been made possible to offer HT with adequate remuneration.

Against the limited general and specific evidence, there is a need to look at predictors or rather factors associated with successful HT compared to inpatient treatment on the level of patient outcomes as well as implementation processes. We therefore used data from the first quasi-experimental evaluation of the German highly regulated model of HT, called inpatient-equivalent treatment (IEHT) or Intensive Home Treatment (IHT) (17), to look at variables associated with successful avoidance or reduction of inpatient psychiatric treatment.

## 2 Methods

### 2.1 Study design and inclusion criteria

The data basis for this evaluation is a multisite, pragmatic, non-randomized quasi-experimental study (called ‘AKtiV’ study) examining the implementation, treatment processes, clinical efficacy, costs as well as user and provider experiences with inpatient-equivalent home treatment in Germany compared to inpatient treatment. The study protocol (18) as well as results of the initial treatment (‘index episode’) (19) and the 12-month-follow up results are published elsewhere (20).

In short, this social health insurance funded study took advantage of ten urban as well as rural hospitals having recently set up IHT teams with a fixed number of places or IHT places associated to special hospital wards or tracks. The study covered a period between 2021 and 2022. All patients having been admitted to IHT were consecutively asked for study participation. Each person participating in the IHT study arm was assigned a propensity score (PS) predicting the probability of receiving this form of treatment in the specific study center. The PS for each potential participant was derived from a logistic regression analysis considering previous hospitalizations, main psychiatric diagnosis, age and gender. For each participant treated via IHT, a match from the pool of recently admitted regular inpatients was built, based on the PS which came closest to the IHT match resulting in the formation of pairs with comparable clinical and sociodemographic characteristics.

Study inclusion criteria for both IHT treated persons as well as for those treated in IT were equal (18) For all participants being allowed to be treated within the German IHT model, additional requirements had to be fulfilled which we did apply also for those asked for participation in the control group (18).

### 2.2 Power calculation

Based on previous HT studies as well as an analysis of readmission rates in the ten study centers, and assuming a 10% drop-out rate, we calculated a sample size of 400 participants necessary to detect a reduction of readmission rates (primary outcome) by 28% from 52.1% (inpatient) to 37.4% (IHT) (two-sided Chi-square test with 5% alpha error and 80% power).

### 2.3 Intervention and control treatment

IHT treatment was provided primarily according to existing standards (17) in the service user’s home by a team of psychiatrists, psychologists, psychiatric nurses, social workers, and further professions at the discretion of the study centers. After recruitment, a psychiatrist assessed IHT suitability, and, together with the team, elaborated a written treatment plan. A consultant in psychiatry had to regularly visit the service user alone or with other team members to assess treatment progress and adjust the treatment procedures. In general, the team had to be available continuously seven days a week. The IHT team met once a week to discuss each participant in detail. The IHT team was responsible for the whole psychiatric care during the crisis, as well as for somatic issues, including diagnostics, medications, psychotherapy, social care, and support. Relatives, informal caregivers, legal guardians, and other persons from the participant’s social network were included as quickly as possible. Participants could use all resources of the hospital. Inpatient treatment of the control group was done according to the standards of psychiatric hospitals in Germany. The control group did not include involuntarily admitted patients or patients with other characteristics which would have prohibited admission to IHT.

### 2.4 Follow-up, assessment and outcome measures

Study staff not involved into delivering care conducted all study assessments. Symptom severity was measured by the German version of the Health of the Nations Outcome Scale (HoNOS-D) (21). The primary outcome measure of the study was inpatient hospital readmission rate within 12 months. In addition to this, we chose “number of days spent in inpatient treatment during the 12-month follow-up period” to evaluate possible predictors for the effectiveness IHT compared with inpatient treatment.

### 2.5 Data analysis

We first selected the following variables used as independent co-variates with a potential influence on the outcomes (re-)admission or number of inpatient days during 12-months-follow-up: (1) Clinician-rated behavioural disturbance, symptom severity, impairment and social functioning as measured by HoNOS value, (2) number of previous hospital stays in the last two years before inclusion in the study, (3) age group, (4) gender (male/female), (5) level of income (numerical), (6) marital status (married or with partner, without partner), and (7) employment (yes/no). As 15 was the HoNOS median within the whole study group, a value of ≤ 15 was interpreted as low (initial) HoNOS and >15 as high.

The aim of the statistical analysis was to test if selected co-variates on the level of participants were associated with the outcome, either as a single predictor variable or within a regression model allowing interactions between each other. In addition, we tested if there was a significant interaction with the treatment group (IHT versus IT), and if the influence of the treatment group on the outcome remained significant after adjusting for the co-variates. The aim was to compare the relative effectiveness of the two treatment models.

First, for each covariate x, a logit regression model was calculated with the regard to the outcome (Y) “admission in the 12-months follow-up time yes/no”. We also calculated an ordinal logistic regression for the variable “number of inpatient hospital days during 12-months-follow-up” using four categories: (a) no (re-)admission/0 days, (b) between 1 and 15 days, (c) between 16 and 32 days, and (d) more than 32 days. For the model with single analyses, each covariate was tested separately according to the equation:


Logit(Y) = constant + a*x + b*group + c*group*x 

where Y is the outcome, group is the treatment group IHT or IT of the index episode, and x is one of the seven co-variates under consideration. The expression group*x indicates the interaction term between the covariate and the treatment group. In addition to the p values, we report Odds Ratios (OR) and their respective 95% Confidence Intervals (95% CI) for the logistic regression in the joint model allowing interaction, to get an impression on the potential clinical relevance.

These single analyses were supplemented for both outcome variables by a joint model where all covariates *x_1,…,_ x_7_* were analyzed together allowing group-interactions. This model follows the equation below:


Logit(Y)=constant + a1*x1 + c1*group*x1 + … + a7*x7 + c7*group*x7 + b*group

The significance level was set at 5%. All tests were explorative.

We used two data sets, the Full Case Analysis (FCA) set and the Multiple Imputation (MI) data set. Both results were compared. The FCA included all study participants with data available. For the MI calculation we generated 100 data sets where missing values were substituted using adequate regression models which included the variance of the outcome variable. For each of the data sets, test statistics were calculated. These statistics were then combined using Rubin’s rules. For each imputed variable we tested if the missing status was associated with the four variables used for PS analysis. In case there were no significant differences we assumed that the value was missing completely at random (MCAR). In this case, imputation was done. We imputed only the outcome variable “admission to inpatient” and not the co-variates.

## 3 Results

### 3.1 Data sets and characteristics of participants

None of the tests to detect a correlation between any PS co-variate and the outcome parameter was significant. Therefore, we assumed that the values were missing completely at random. For the readmission rate, we had only 6.25% missing values.

200 received IHT and 200 IT. Both groups were comparable with regard to clinical as well as sociodemographic variables. Two thirds of the whole sample (n=264; 66%) were female, the mean age was 45 (SD = 19) years. Most participants had a diagnosis of recurrent depression or a depressive or manic episode within a bipolar disorder (n = 94; 47%), a psychotic episode (n = 43; 21.5%), an episode of an anxiety disorder, an obsessive-compulsory disorder or a somatoform disorder (n = 31; 15.5%) or a personality disorder (n = 16; 8%). The mean number of psychiatric IT or IHT episodes during the last two years was 1.6 in the IHT group and 1.2 in the IT group. Most participants were single (n = 107; 53.5%) in the IHT and 64.5% (n = 129) in the IT group) with only between 25.5% (IHT, n = 51) and 32.5% (IHT, n = 65) working competitively in the formal labour market.

Symptom severity as measured by HoNOS was moderate and comparable between both groups (14.7 in the IHT groups and 15.3 in the IT group). There were no significant differences in clinical parameters between the two groups.

### 3.2 Factors associated with (re-)admission to inpatient treatment

We tested center effects and found no center effect and no significant variability among centers. Therefore, we did not include center as a control variable.

In the single analyses, only the HoNOS value had a significant influence on the readmission rate in both treatment groups, with higher values being associated with a higher risk (p co-variate). After including the co-variates, the difference between the groups with regard to the readmission risk remained significant with the exception of age and gender (**Table 1**).

**Table 1:** Possible factors associated with (re-)admission, Intensive Home Treatment vs. Inpatient Treatment.

Variable	Single analyses			Joint model (p group: 0.7468)		
	*p* co-variate	*p* interaction	*p* group (IHT vs IT)	Odds Ratio (95%CI)	*p* co-variate	*p* interaction
Severity (HoNOS value)	0.0462*	0.1438	0.0117*	0.94 (0.85 – 1.06)	0.2120	0.3139
Number of previous hospital stays	0.1095	0.8669	0.0006*	0.87 (0.61 – 1.22)	0.0290*	0.4119
Age group (young/old)	0.6095	0.5241	0.5888	2.78 (0.85 - 9.07)	0.1304	0.0892
Gender (female/male)	0.0730	0.0659	0.4478	3.75 (1.16 – 12.14	0.1028	0.0280*
Net income (numeric)	0.4907	0.8759	0.0050*	0.99 (0.98 – 1.01)	0.5266	0.2932
Marital status (yes/no)	0.3192	0.9315	0.0104*	1.38 (0.38 – 4.96)	0.6944	0.6217
Employment status (yes/no)	0.4945	0.8432	0.0025*	0.69 (0.20 – 2.33)	0.7639	0.5463

CI, Confidence Interval; HoNOS, Health of the Nations Outcome Scale; IHT, Intensive Home Treatment; IT, Inpatient Treatment; * = statistically significant at p<0.05.

In the analytical model with all co-variates included and allowing interactions between co-variates and the outcome variable, we found that only the number of previous hospital-based treatment episodes had a significant positive influence on readmission rates. However, taking into account all co-variates, women had a significantly lower risk to be admitted to the hospital after IHT compared to initial IT. These results did not change significantly when using the MI data set.

### 3.3 Factors associated with the amount of inpatient hospital days during follow-up

Three of the seven co-variates interacted with the outcome variable “inpatient hospital days” in the ordinal regression model (**Table 2**): severity as measured by HoNOS, number of previous hospital stays, and gender. In the model with all co-variates included, only the number of previous hospital stays was significant. We found no significant association between predictor variables and the outcome.

**Table 2:** Possible factors associated with the number of inpatient days, Intensive Home Treatment vs. Inpatient Treatment.

Variable	Single analyses			Joint model (p group: 0.7581)	
	*p* co-variate	*p* interaction	*p* group (IHT vs IT)	*p* co-variate	*p* interaction
Severity (HoNOS value)	0.0147*	0.0454*	0.0042*	0.0915	0.1218
Number of previous hospital stays	0.0361*	0.7098	0.0009*	0.0149*	0.5050
Age group (young/old)	0.1221	0.1074	0.6103	0.0590	0.0527
Gender (female/male)	0.0466*	0.0702	0.4076	0.1871	0.1154
Net income (numeric)	0.2012	0.5532	0.1143	0.8732	0.6144
Marital status (yes/no)	0.3095	0.8058	0.0244*	0.8764	0.8802
Employment status (yes/no)	0.4867	0.8804	0.0134*	0.5065	0.5434

HoNOS, Health of the Nations Outcome Scale; IHT, Intensive Home Treatment; IT, Inpatient Treatment; * = statistically significant at p<0.05.

Participants in IHT with low initial HoNOS (≤ 15), spent an average of 10.3 days (SD = 33.8) in the hospital during the 12-months follow-up period, whereas those with high severity (HoNOS > 15) spent 19.6 days (SD = 40.2). Participants using initial IT with low baseline HoNOS spent an average of 20.7 days (SD = 38.8) in the hospital during the 12 months follow-up period, whereas those with high severity spent 21.5 days (SD = 45.4).

Participants in IHT without any hospital stays in the last two years, spent an average of 6.3 days (SD = 24.7) in hospital during the 12-months follow-up period, whereas those with at least one previous hospital stay spent 20.9 days (SD = 43.5). The results for those treated initially in an inpatient setting were as follows: 8.2 days (SD = 19.5) in those without any hospital stays in the last two years versus 37.5 days (SD = 55.5) in those with at least one previous hospital stay.

Female participants in IHT spent an average of 11 days (SD = 31.0) in hospital, whereas men spent 21.3 days (SD = 46.4). In the IT group, female participants spent an average of 21.5 days (SD = 42.5) in hospital, whereas male spent 20.3 days (SD = 41.6).

## 4 Discussion

### 4.1 Main findings

In our study, IHT seemed to be more effective in women than in men to prevent readmission. The same was true for the time spent in inpatient treatment: IHT could not reduce time spent in hospital in men, whereas women spent less time in hospital after they had been treated by IHT. In addition, in people with less impairment and those with no inpatient history or a high number of previous hospitalizations in the last two years, IHT was more effective in preventing rehospitalization.

### 4.2 Other studies

In a Swiss study, similar to our study, two thirds of HT patients were female with more having affective than psychotic disorders (22). In patients who were less severely ill and those with a better level of social functioning indicated e.g. by formal employment and hospital admissions, hospital admissions could be more successfully avoided by HT than with other patients. However, there was no gender effect in the Swiss study.

In a Dutch study, predictors were tested with regard to the level of psychiatric symptoms as measured with the Brief Psychiatric Rating Scale (BPRS) after 26 and 52 months (23). The authors found an effect of the employment status after 26 weeks, but not after 52 weeks. Being unemployed increased the risk of higher symptom severity score after 26 weeks. Age, gender, domestic situation, or type of diagnosis had no moderating effect on the symptom level at follow-up. With regard to the relative effectiveness of HT versus care as usual on psychiatric symptoms, the authors found no convincing evidence for any predictor thus HT was recommended to be offered to a diverse target population with comparable clinical results.

A recent systematic review also found that high symptom severity and former hospital admissions were associated with unsuccessful treatment outcome in HT (24). Our study could not replicate the finding that formal employment and regular income were associated with a positive outcome. We did not formally measure family involvement which was one of the success factors in the review (24).

### 4.3 Explanatory approaches

Our results on a differential effect of HT compared to IT according to gender raises questions on whether and why psychiatric treatment outside the hospital is in general more effective in women than in men. As our results are exploratory and other moderators were not directly measured we can only hypothesize about possible explanations for this result. One might ask if the relative effectiveness of IHT in women was because inpatient treatment is often not able to meet female patients’ needs or rather that HT might be more suitable for women. Some studies show that women with psychosis in general may benefit more from psychiatric treatment, and that they have a better course than men (25). This might be, however, true for the whole life course and not for a single episode, and maybe valid independent of the treatment modality. In contrast to this, there is no clear evidence that women with depression might have a higher or lower risk of recurrence or might benefit more from depression treatment than men. The differences in symptoms and comorbid psychopathology between men and women are generally related to a help-seeking selection bias, but these differences did not translate into differential outcomes (26).

One explanation for the higher relative effectiveness of IHT in women might be women’s higher preference to be treated in their social environment to get more social and emotional support in their own context resulting in a more active role during HT. It is well known that women tend to have larger social networks than men (27, 28). It might be easier within HT to build on these networks in women thus increasing their coping mechanisms more effectively than in men. In general, in further evaluations of HT approaches, family involvement should be explicitly measured at baseline and evaluated during follow-up with suitable instruments such as the Social Network Schedule (SNS) or others (29). On the one hand, building upon existing social networks might be more easily possible with HT compared to inpatient treatment during severe psychological crises. On the other hand, re-connecting to the family and other social networks might be a relevant outcome parameter of a specific treatment modality.

In our study, patients with no previous hospital stays and those with less severe symptoms seem to benefit more from IHT. Avoiding inpatient treatment may be of higher significance in this group of people as the experience of being able to cope with a psychiatric crisis at home without hospitalization translates in greater self-efficacy and confidence to deal with future crises. A Dutch HT study, however, could not show that HT has a supplementary effect on self-efficacy when compared to treatment as usual, although self-efficacy was associated with symptomatic recovery and quality of life, and patients with depression increased their self-efficacy between baseline and both six and 26 weeks (30).

Together with the significant superiority compared to inpatient treatment, the lack of clear predictors for HT effectiveness might be an indicator either for the broad effectiveness of this treatment modality, or a lack of suitability of current diagnostic, clinical or sociodemographic characterizations of people in severe psychiatric crises. This situation requires more research efforts.

### 4.4 Limits

As our study was not randomized, we have no solid evidence how many people in IT could have been treated with IHT without inpatient admission. As all of our patients participated voluntarily there is some risk of self-selection, although both IHT as well as the control group had similar values in clinical severity and utilization patterns. In general, there is always some risk of a residual selection bias as patients willing to receive home treatment may differ from those receiving inpatient care in important unmeasured characteristics such as treatment preference, family support, motivation, insight, or willingness to engage with community-based services. These factors may influence both treatment allocation and outcomes. Potentially important clinical and social variables (such as employment and marital status) were not included in the propensity score calculation as they were not available at the time of propensity score matching, despite having a potential influence on the decision to refer a patient to IHT or not. For example, the decision to take part in IHT may be an indicator of a stronger preference not to be admitted to hospital.

We did use only utilization and no clinical data to measure the relative effectiveness of IHT. Readmission rates and hospital days are utilization data which are just indicators of clinical events such as symptom severity of functional recovery. Readmission might also be influenced by systemic factors (such as pressure for bed occupancy or clearance, lack of social support at home). Conversely, patients may experience severe clinical deterioration at home without being readmitted (due to patient refusal or system failure). Thus, readmission does not equal clinical worsening. However, in our opinion, readmission is a valid outcome indicator for a variety of diagnoses as there are, in general, few readmissions without worsening of the disorder or relapse or recurrence (31).

Another limitation is the fact that our study was not specifically powered to detect interaction effects. Subgroup and interaction analyses lacking adequate statistical power carry a higher risk of Type II error which has to be taken into account when interpreting the findings.

## 5 Conclusion

Our result that women might have a higher chance to avoid hospitalization after IHT could indicate either that IHT is more ‘adequate’ for them, that they feel more comfortable at home, that they can build on more resources at home, or that they benefit less than men from IT in general. The finding that people with more severe symptoms or longer treatment histories may benefit less from intensive HT should not lead to these people being offered this treatment modality less in current practice. Good everyday experience has shown that these people can also be helped well, for example with multiple treatment episodes in HT with breaks in between.

This study represents a further step in understanding the differential effectiveness of HT compared with IT for various patient subgroups. Our research highlights the impact of gender on HT effectiveness, favoring women. However, further research is needed to refine our understanding of the mechanisms behind HT’s effectiveness and to develop more nuanced predictors for personalized treatment selection, considering individual patient characteristics and gender. The current results will inform modifications to our research methodology, incorporating additional variables and refining outcome measures to enhance the validity and generalizability of findings.

## Data Availability

The raw data supporting the conclusions of this article will be made available by the authors, without undue reservation.

## References

[1] Johnson S Needle J Bindman J Thornicroft G . Crisis Resolution and Home Treatment in Mental Health. Cambridge, United Kingdom: Cambridge University Press (2007).

[2] Johnson S Nolan F Pilling S Sandor A Hoult J McKenzie N . Acute psychiatric care: Approaches to increasing the range of services and improving access and quality of care. World Psychiatry: Off J World Psychiatr Assoc (WPA). (2022) 21:220–36. doi: 10.1002/wps20962 35524608 PMC9077627

[3] Department of Health and Social Care (DHSC) . The NHS Plan: A Plan for Investment a Plan for Reform. London, United Kingdom: DHSC (2000).

[4] McGlynn P . Crisis Resolution and Home Treatment a Practical Guide. London, United Kingdom: The Sainsbury Centre for Mental Health (2006). Available online at: https://wwwuclacuk/core-study/sites/core-study/files/mcglynn_2006_Crisis_resolution_and_home_treatment_guidepdf (Accessed July 12, 2026).

[5] Stulz N Wyder L Maeck L Hilpert M Lerzer H . Home treatment for acute mental healthcare: Randomised controlled trial. Br J Psychiatry: J Ment Sci. (2020) 216:323–30. doi: 10.1192/bjp201931 30864532

[6] Cornelis J Barakat A Blankers M Peen J Lommerse N . The effectiveness of intensive home treatment as a substitute for hospital admission in acute psychiatric crisis resolution in the Netherlands: A two-centre Zelen double-consent randomised controlled trial. Lancet Psychiatry. (2022) 9:625–35. doi: 10.1016/S2215-0366(22)00187-0 35779532

[7] Joy CB Adams CE Rice K . Crisis intervention for people with severe mental illnesses. Cochrane Database Systematic Rev. (2006) 4:CD001087. doi: 10.1002/14651858CD001087pub3 17054133

[8] Lloyd-Evans B Christoforou M Osborn D Ambler G Marston L . Crisis Resolution Teams for People Experiencing Mental Health Crises: The CORE Mixed-Methods Research Programme Including Two Rcts. Southampton, United Kingdom: NIHR Journals Library. (2019). doi: 10.3310/pgfar07010. 30986008

[9] Lloyd-Evans B Osborn D Marston L Lamb D Ambler G . The CORE service improvement programme for mental health crisis resolution teams: Results from a cluster-randomised trial. Br J Psychiatry: J Ment Sci. (2020) 216:314–22. doi: 10.1192/bjp201921 30761976 PMC7511901

[10] Hoult J Reynolds I Charbonneau-Powis M Weekes P Briggs J . Psychiatric hospital versus community treatment: The results of a randomised trial. Aust New Z J Psychiatry. (1983) 17:160–7. doi: 10.3109/00048678309160000 6578788

[11] Hasselberg N Gråwe RW Johnson S Šaltytė-Benth J Ruud T . Psychiatric admissions from crisis resolution teams in Norway: A prospective multicentre study. BMC Psychiatry. (2013) 13:117. doi: 10.1186/1471-244X-13-117 23594922 PMC3637541

[12] Johnson S Nolan F Pilling S Sandor A Hoult J McKenzie N . Randomised controlled trial of acute mental health care by a crisis resolution team: the North Islington crisis study. BMJ. (2005) 331(7517):599. doi: 10.1136/bmj.38519.678148.8F 16103032 PMC1215550

[13] Pelosi AJ Jackson GA . Home treatment—Enigmas and fantasies. BMJ (Clinical Res Ed). (2000) 320:308–9. doi: 10.1136/bmj.320.7230.308 10722288

[14] Smyth MG Hoult J . The home treatment enigma. BMJ (Clinical Res Ed). (2000) 320:305–8. doi: 10.1136/bmj3207230305 10650032 PMC1117491

[15] European Commission . State of Health in the EU - Synthesis Report. Luxembourg: Publications Office of the European Union (2023). Available online at: https://healtheceuropaeu/system/files/2023-12/state_2023_synthesis-report_enpdf (Accessed July 12, 2026).

[16] von Peter S Ignatyev Y Johne J Indefrey S Kankaya OA Rehr B . Evaluation of flexible and integrative psychiatric treatment models in Germany-a mixed-method patient and staff-oriented exploratory study. Front Psychiatry. (2018) 9:785. doi: 10.3389/fpsyt201800785 30723433 PMC6349706

[17] GKV-Spitzenverband und Deutsche Krankenhausgesellschaft . Vereinbarung Zur Stationsäquivalenten Psychiatrischen Behandlung Nach § 115d Abs 2 SGB V Vom 01082017 (2017). Available online at: https://wwwgkv-spitzenverbandde/media/dokumente/krankenversicherung_1/krankenhaeuser/psychiatrie/2017_08_01_KH_Vereinbarung_StaeB_115_d_Abs_2_SGB_V_Unterschriftenfassungpdf (Accessed July 12, 2026).

[18] Baumgardt J Schwarz J Bechdolf A Nikolaidis K Heinze M . Implementation efficacy costs and processes of inpatient equivalent home-treatment in German mental health care (AKtiV): Protocol of a mixed-method participatory quasi-experimental trial. BMC Psychiatry. (2021) 21:173. doi: 10.1186/s12888-021-03163-9 33781237 PMC8008509

[19] Weinmann S Nikolaidis K Längle G von Peter S Brieger P . Premature termination satisfaction with care and shared decision-making during home treatment compared to inpatient treatment: A quasi-experimental trial. Eur Psychiatry. (2023) 66:e71. doi: 10.1192/jeurpsy20232443 37681407 PMC10594305

[20] Bechdolf A Nikolaidis K von Peter S Längle G Brieger P . Utilization of psychiatric hospital services following intensive home treatment: A non-randomized clinical trial. JAMA Network Open. (2024) 7(11):e2445042. doi: 10.1001/jamanetworkopen202445042 39546314 PMC11568461

[21] Fankhauser S Hochstrasser B Sievers M Soyka M . Assessing change of depressive symptoms and severity of depression in an inpatient setting: Performance of the HoNOS (Health of the Nation Outcome Scales). Psychotherapie Psychosomatik Medizinische Psychol. (2017) 67:391–400. doi: 10.1055/s-0043-105482 28718866

[22] Mötteli S Jäger M Hepp U Wyder L Vetter S . Home treatment for acute mental healthcare: Who benefits most? Community Ment. Health J. (2021) 57:828–35. doi: 10.1007/s10597-020-00618-3 32279118

[23] Barakat A Blankers M Cornelis JE Lommerse NM Beekman AT . Prescriptive factors for intensive home treatment in acute psychiatry: A secondary analysis of a randomised controlled trial. Int J Ment Health Syst. (2024) 18:3. doi: 10.1186/s13033-023-00619-1 38172935 PMC10763431

[24] Bergamaschi V Baumann F Warnke I Corbisiero S Ludwig F . Who benefits from acute psychiatric home treatment? A systematic review. Community Ment Health J. (2024). doi: 10.1007/s10597-024-01297-0 38940978 PMC11408559

[25] Seeman MV . Does gender influence outcome in schizophrenia? Psychiatr Q. (2019) 90:173–84. doi: 10.1007/s11126-018-9619-y 30484001

[26] Weissman MM . Treatment of depression: Men and women are different? Am J Psychiatry. (2014) 171:384–7. doi: 10.1176/appiajp201313121668 24687191

[27] Ang S . Life course social connectedness: Age-cohort trends in social participation. Adv Life Course Res. (2019) 39:13–22. doi: 10.1016/j.alcr.2019.02.002 38826717

[28] Milner A Krnjacki L LaMontagne AD . Age and gender differences in the influence of social support on mental health: A longitudinal fixed-effects analysis using 13 annual waves of the HILDA cohort. Public Health. (2016) 140:172–8. doi: 10.1016/j.puhe.2016.06.029 27527844

[29] Degnan A Berry K Sweet D Abel K Crossley N . Social networks and symptomatic and functional outcomes in schizophrenia: A systematic review and meta-analysis. Soc Psychiatry Psychiatr Epidemiol. (2018) 53:873–88. doi: 10.1007/s00127-018-1552-8 29951929 PMC6133157

[30] Barakat A Blankers M Cornelis JE Lommerse NM Beekman ATF . The effects of intensive home treatment on self-efficacy in patients recovering from a psychiatric crisis. Int J Ment Health Syst. (2021) 15:2. doi: 10.1186/s13033-020-00426-y 33407731 PMC7789166

[31] Fischer C Lingsma HF Marang-van de Mheen PJ Kringos DS Klazinga NS . Is the readmission rate a valid quality indicator? A review of the evidence. PLoS ONE. (2014) 9(11):e112282. doi: 10.1371/journal.pone.0112282 25379675 PMC4224424

